# Early single-channel aEEG/EEG predicts outcome in very preterm infants

**DOI:** 10.1111/j.1651-2227.2012.02677.x

**Published:** 2012-07

**Authors:** Sverre Wikström, Ingrid Hansen Pupp, Ingmar Rosén, Elisabeth Norman, Vineta Fellman, David Ley, Lena Hellström-Westas

**Affiliations:** 1 Department of Women's and Children's Health, Uppsala UniversityUppsala, Sweden; 2 Center for Clinical Research, County Council of VärmlandKarlstad, Sweden; 3 Division of Pediatrics, Department of Clinical Sciences, Lund University HospitalLund, Sweden; 4 Department of Clinical Neurophysiology, Lund University HospitalLund, Sweden; 5 Children′s Hospital, University of HelsinkiHelsinki, Finland

**Keywords:** Burst suppression, Cranial ultrasound, Interburst interval, Neurodevelopmental impairment, Seizure

## Abstract

**Aim:**

To characterize early amplitude-integrated electroencephalogram (aEEG) and single-channel EEG (aEEG/EEG) in very preterm (VPT) infants for prediction of long-term outcome.

**Patients:**

Forty-nine infants with median (range) gestational age of 25 (22–30) weeks.

**Methods:**

Amplitude-integrated electroencephalogram/EEG recorded during the first 72 h and analysed over 0–12, 12–24, 24–48 and 48–72 h, for background pattern, sleep–wake cycling, seizures, interburst intervals (IBI) and interburst percentage (IB%). In total, 2614 h of single-channel EEG examined for seizures. Survivors were assessed at 2 years corrected age with a neurological examination and Bayley Scales of Infant Development-II. Poor outcome was defined as death or survival with neurodevelopmental impairment. Good outcome was defined as survival without impairment.

**Results:**

Thirty infants had good outcome. Poor outcome (n = 19) was associated with depressed aEEG/EEG already during the first 12 h (p = 0.023), and with prolonged IBI and higher IB% at 24 h. Seizures were present in 43% of the infants and associated with intraventricular haemorrhages but not with outcome. Best predictors of poor outcome were burst-suppression pattern [76% correctly predicted; positive predictive value (PPV) 63%, negative predictive value (NPV) 91%], IBI > 6 sec (74% correctly predicted; PPV 67%, NPV 79%) and IB% > 55% at 24 h age (79% correctly predicted; PPV 72%, NPV 80%). In 35 infants with normal cerebral ultrasound during the first 3 days, outcome was correctly predicted in 82% by IB% (PPV 82%, NPV 83%).

**Conclusion:**

Long-term outcome can be predicted by aEEG/EEG with 75–80% accuracy already at 24 postnatal hours in VPT infants, also in infants with no early indication of brain injury.

## Introduction

The high prevalence of neurodevelopmental impairment (NDI) after very preterm (VPT) birth is still a concern (1). The aetiology for NDI is multifactorial and includes acute brain damage, such as germinal matrix and intraventricular haemorrhages (IVH) occurring during the first postnatal days, as well as white matter damage that may develop later during the neonatal period.

Development of methods that enable early identification of acute brain injury in preterm infants is urgent, not only to direct overall care and inform parents about their infant's condition, but also to obtain predictive information as a basis for neuroprotective intervention. The electrocortical background pattern (BG) in the amplitude-integrated electroencephalogram (aEEG)/EEG has proved to be one of the best early predictors of outcome in term asphyxiated infants (2). However, less is known about the long-term predictive value of early aEEG/EEG in VPT infants (3–6).

The normal EEG background in VPT infants is mainly discontinuous (DC), characterized by high-voltage activity bursts and interburst intervals (IBI) of low voltage. We and others have shown that the highest burst rate per hour (i.e. best background) in aEEG recorded at 24–48 postnatal hours was predictive of outcome in preterm infants with large IVH (grade 3–4) (4). Further, IBIs averaged over the first 72 postnatal hours were associated with outcome; VPT infants developing handicaps had significantly longer IBI than infants without handicaps (7). A neurophysiologist's assessment regarding IBI, waveforms and seizures in a two-channel EEG recorded from VPT infants within 48 postnatal hours resulted in a positive predictive value (PPV) of 75% and a negative predictive value (NPV) of 88% for adverse outcome at 15 months, respectively (5).

**Key notes**

- In very preterm infants, the early aEEG/EEG background, as recorded at 24–48 postnatal hours, is predictive of outcome with around 80% accuracy, indicating the impact of early brain damage on long-term outcome.
- Brief subclinical seizures are common during the first postnatal days and are associated with IVH, but not long-term outcome.

With the hypothesis that higher proportions of discontinuous electro-cortical background (i.e. suppressed aEEG/EEG) reflect an unfavourable prognosis, the objectives of this study were to characterize early aEEG/EEG development in VPT infants in relation to outcome at 2 years of age; to assess the predictive value of aEEG/EEG on long-term outcome; and to assess the value of a one-hour high-quality recording as compared to the long-term aEEG/EEG trend.

## Patients and Methods

### Patients

Fifty-four infants were prospectively included in the study between July 2005 and May 2007 after antenatal written parental consent; 45 had a gestational age (GA) <28 weeks (extremely preterm, EPT), and nine were born at 28–30 weeks GA (VPT). GA was determined by prenatal ultrasound at 17–18 weeks. All infants were treated at the tertiary level neonatal intensive care unit (NICU) at Lund University Hospital, Sweden. The final study cohort consisted of 49 infants; 41 had follow up at 2 years of corrected age and eight infants who died. Five surviving infants were excluded because two children moved prior to follow-up, and three families declined further participation. The Regional Ethical Review Board at Lund University approved the protocol.

### Clinical settings

The infants underwent standard NICU care and had an additional recording of a single-channel EEG during the first 72 postnatal hours. Bolus doses of morphine (most often 0.05–0.15 mg/kg), or continuous infusion of morphine, were administered on clinical indications to mechanically ventilated infants. Thirty infants received in median (range) 3 (1–7) bolus doses of morphine during the study period. In six of these infants, morphine was at some time administered as infusion. Repeated cerebral ultrasound investigations (cUS) were performed (Acuson Sequoia, 8.5 MHz; Siemens Healthcare, Erlangen, Germany) on days 1, 3 and 7 and at 3 and 6 weeks, and at term. Germinal matrix and IVH were classified according to Papile (8). Periventricular leukomalacia was defined as periventricular echodensities persisting for more than 7 days, or periventricular cysts (9). The predictive value of early aEEG was assessed separately in the subgroup of infants with normal cUS findings during the first 3 days.

### Single-channel aEEG/EEG

A single-channel EEG recording with the aEEG trend (Nervus monitor, 1.3 EEG System; Taugagreining HF, Reykjavik, Iceland) was initiated as soon as possible after initial stabilization (median 8 h). After gentle skin preparation (Nuprep Skin Prep Gel™; Weaver and Company, Aurora, CO, USA), hydrogel electrodes (Ambu® Neuroline; Ambu A/S, Ballerup, Denmark) were applied at P3 and P4, according to the International 10–20 System, as well as a frontal reference.

**Figure 1:**
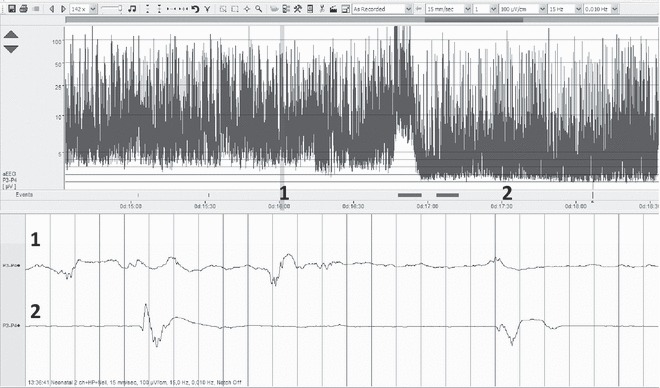
Four hours of [amplitude-integrated electroencephalogram (aEEG)] trend with corresponding 25 sec of single-channel EEG below from two different time-points, displaying discontinuous (DC) pattern (1) and burst suppression (2). This 4-h epoch would obtain a score of 3 (see text), because >50% of the recording was DC. No cyclicity is evident.

Two investigators (SW and LHW, together), blinded for the patient's identity and clinical data, assessed visually the aEEG trend, displayed at 6 cm/h, in 4-h epochs. For each epoch, the dominating (>50%) aEEG BG was scored in six categories using a published scoring system (10): 0 = flat/inactive, 1 = sparse burst suppression (BS), 2 = dense BS, 3 = mixed BS and DC, 4 = DC, 5 = continuous (C). For each epoch, sleep–wake cycling (SWC) was scored: 0 = no cyclicity, 1 = imminent cyclicity, 2 = immature cyclicity, 3 = developed SWC. The aEEG assessment was repeatedly verified by the inspection of the original single-channel EEG, Figure 1.

Interburst intervals were quantified by using a validated automated segmentation algorithm based on a non-linear energy operator (NLEO), reflecting both amplitude and frequency content of the EEG (11,12). Before the IBI analysis, parts containing interference or seizures were excluded from analysis because they were expected to affect the NLEO-algorithm. The percentage of recording detected as IBI was called interburst percentage (IB%).

Median values of consecutive 4-h epochs of each infant's BG score, SWC score, IBI and IB%, respectively, were calculated over the first 0–12, 12–24, 0–24, 24–48 and 48–72 postnatal hours.

For a comparison of continuous and intermittent aEEG/EEG, IBI and IB% were quantified in a 1-h high-quality single-channel EEG selected from each recording as close as possible after 24-h age while meeting the following criteria: No artefacts identified in the original EEG; no handling of the infant; and no morphine (13) administered during the previous four hours. If SWC was present, the 1-h epoch was selected ±30 min from maximal continuity (corresponding to wakefulness or active sleep).

Electrographic seizures were identified in the single-channel EEG by an experienced neurophysiologist (IR) who examined all recordings (in total 2614 h) at 10 mm/sec and 70 μV/cm sensitivity. A seizure was defined as an abnormal electrical event, lasting for 10 sec or more, with evolving, repetitive waveforms with a gradual build-up and reaching a maximum before declining in frequency, morphology or amplitude (14). The duration of each seizure was measured. Thereafter, the corresponding aEEG trend epoch was analysed for the presence of seizure activity. The aEEG/EEG recordings were not assessed, and seizures were neither recognized nor treated during clinical care.

### Follow-up

Neurodevelopmental outcome assessed at 2 years corrected age included a standardized neurological examination and Bayley Scales of Infant Development-II (BSID-II) with the two subscales Mental Developmental Index (MDI) and Psychomotor Developmental Index (PDI) (15). NDI was defined as presence of cerebral palsy, or MDI < 70, or PDI < 70, or blindness, or deafness. Good outcome was defined as surviving without NDI, and poor outcome was defined as death or survival with NDI.

### Statistical analysis

Non-parametric statistics were used for group comparisons and analysis of changes over time in aEEG/EEG measures. Cerebral ultrasound findings during the first three postnatal days and the following aEEG/EEG measures were evaluated as predictors of outcome: background scores, SWC scores, IBI and IB%. Sensitivity, specificity, PPV, NPV and percentage of correctly predicted infants were calculated. Receiver operating characteristics (ROC) curves, with area under the curve (AUC) estimations, were produced for identification of optimal cut-offs. Odds ratios for poor outcome were calculated in logistic regression models, adjusting for GA.

## Results

The median (range) duration of the aEEG/EEG recordings was 56 (14–71) h, with 32 (7–64) h per infant having sufficient technical quality for analysis of IBI and IB% (in two infants, the quality of the EEG recording only allowed for visual analysis). Follow-up at 2 years was conducted in 41 infants; eight VPT and 22 EPT infants survived with good outcome, while 11 EPT infants survived with NDI. Eight EPT infants died in the neonatal period, that is, in total, 19 EPT infants had a poor outcome. Clinical data are presented in Table 1.

**Table 1:** Clinical data of the study group presented according to gestational age (GA) group and long-term outcome. Good outcome defined as survival without neurodevelopmental impairment (NDI), and poor outcome defined as death or survival with NDI

	EPT, poor outcome n = 19	EPT, good outcome n = 22	VPT, good outcome n = 8
GA, weeks	25 (22–27)	25 (23–27)	28 (28–30)**
Birth weight, gram	740 (440–980)	814 (520–1148)	1288 (1137–1716)
5-min Apgar score	6 (2–10)	8 (3–10)	8 (4–10)
Male/female, n	11/8	10/12	5/3
IVH grade I–II < 7 day, n	2	7	0
IVH grade III–IV < 7 day/PVL, n	6*	1	1

Values are medians (ranges) or frequencies.

* p < 0.05 difference between EPT infants with good versus poor outcome.

** p < 0.01 difference between EPT infants with good outcome versus VPT infants.

EPT = extremely preterm; IVH = intraventricular haemorrhages; PVL = periventricular leukomalacia; VPT = very preterm.

### Visual and quantitative assessment of the aEEG/EEG background

**Figure 2:**
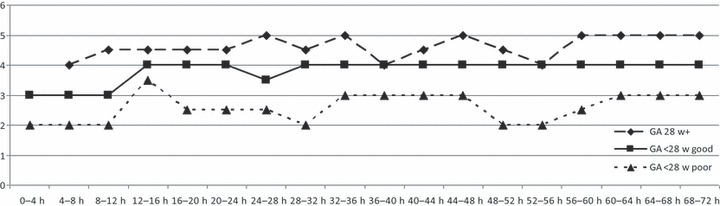
Median background scores in each individual 4-h epoch for each outcome category (EPT poor outcome, EPT good outcome, VPT good outcome). Significant statistical differences (p < 0.05) in background patterns between EPT infants with poor versus good outcome first occurred at 16–20 h. EPT, extremely preterm; VPT, very preterm.

The aEEG/EEG background scores were significantly lower in infants with poor outcome at 0–12, 12–24, 24–48 and 48–72 h as compared to those with good outcome (p = 0.023, p = 0.036, p ≤ 0.001, and p = 0.003, respectively). Median IBI and IB% were higher in infants with poor outcome at 24–48 and 48–72 h (IBI p = 0.004 and p = 0.009; IB% p = 0.004 and p = 0.002). Background scores, IBI and IB%, differed significantly at 24–48 and 48–72 h between EPT infants with poor and good outcome (Tables 2 and 3, Fig. 2).

**Table 2:** Summary of aEEG/EEG results during the first 72 postnatal hours and according to gestational age and outcome, including visual assessment of background and sleep–wake cycling (SWC) scores and quantitative measures of interburst interval (IBI) and interburst percentage (IB%)

Time period	aEEG/EEG	EPT poor outcome n = 19	EPT good outcome n = 22	VPT good outcome n = 8
0–24 h	Background	2.5 (1–4)	3.5 (1–5)	4 (3–5)
	SWC	0 (0–1)	0 (0–2)	0.5 (0–1)
	IBI, sec	7.1 (5.0–16.0)	6.3 (3.7–12.8)	6.7(4.3–12.6)
	IB%	57.6 (31.7–84.7)	48.7 (26.9–79.0)	54.8 (28.5–77.9)
24–48 h	Background	2.75 (1–4)†	4 (2–5)	4.75 (2–5)**
	SWC score	0 (0–1)*	0 (0–2)	0 (0–1)
	IBI, sec	6.8 (4.4–14.4)†	5.2 (3.1–8.4 9.8)	3.9 (2.4–5.9)**
	IB%	58.1 (23.9–79.4)†	41.3 (15.8–68.9)	28.5 (21.6–46.9)
48–72 h	Background	3 (1.5–5)*	4 (2–5)	5 (3.5–5)
	SWC	0.25 (0–2)	1 (0–2.5)	1 (0–1)
	IBI, sec	6.3 (3.8–8.5)*	4.7 (3.6–8.8)	4.1 (3.0–4.4)**
	IB%	56.8 (25.6–69.4)*	35.6 (20.4–68.3)	21.3 (14.4–28.8)††

Values are medians (ranges).

* p < 0.05 difference between EPT infants with good versus poor outcome.

** p < 0.05 difference between EPT infants with good outcome versus VPT infants.

† p < 0.01 difference between EPT infants with good versus poor outcome.

†† p < 0.01 difference between EPT infants with good outcome versus VPT infants.

aEEG = amplitude-integrated electroencephalogram; EPT = extremely preterm; VPT = very preterm.

**Table 3:** aEEG/EEG results during the first 24 h according to gestational age and outcome, including visual assessment of background and sleep–wake cycling (SWC) scores and quantitative measures of interburst interval (IBI) and interburst percentage (IB%)

Time period	aEEG/EEG	EPT poor outcome n = 19	EPT good outcome n = 22	VPT good outcome n = 8
0–12 h	Background	2 (1–5)*	3 (2–5)	4.25 (4–5)
	SWC	0 (0–0.5)	0 (0–2.5)	0 (0–0)
	IBI, sec	6.9 (4.9–15.3)	6.5 (2.6–14.1)	7.0 (3.7–7.5)
	IB%	58 (39–82)	44 (26–80)	56 (23–57)
12–24 h	Background	2.5 (1–4)	4 (1–5)	4 (2–5)
	SWC	0 (0–1)	0 (0–2)	1 (0–1)
	IBI, sec	7.0 (5.0–18.1)	5.8 (3.1–12.8)	5.6 (4.3–9.6)
	IB%	58 (32–89)	54 (27–79)	43 (32–63)
1–h at 24 h	IBI, sec	7.4 (4.9–35.4)†	5.5 (2.5–9.5)	5.1 (2.3–17.7)
	IB%	61.5 (35.0–92.1)†	41.1 (19.9–77.1)	28.4 (0–82.2)

Values are medians (ranges).

* p < 0.05 difference between EPT infants with good versus poor outcome.

† p < 0.01 difference between EPT infants with good versus poor outcome.

aEEG = amplitude-integrated electroencephalogram; EPT = extremely preterm; VPT = very preterm.

**Figure 3:**
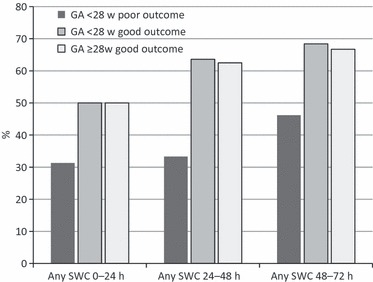
Percentage of infants with any SWC present in relation to time after birth, gestational age and long-term outcome. SWC, sleep–wake cycling.

Continuous activity (BG score 5) was present in 53% of infants with good outcome (p = 0.002 vs poor outcome) and was identified in 88% of the VPT infants, in 41% of the EPT infants with good outcome but only in 11% of EPT infants with poor outcome (p = 0.038 for EPT comparison). BS dominated at least one 4-h epoch in 84% of infants with poor outcome, but only in 33% of infants with good outcome (p = 0.001). BS was present in 25% of VPT infants, in 84% of EPT infants with poor outcome and in 36% of EPT infants with good outcome (p = 0.004 for EPT comparison). SWC was identified during the first 72 h in 64% of the infants, more often in infants with good outcome (p = 0.029), Figure 3. At 24–48 h, the highest score for SWC differed significantly between infants with good versus poor outcome (p = 0.022).

In infants with good outcome, the median BG and SWC scores increased between 0–24 and 24–48 h (p = 0.020 and p = 0.034, respectively), and median IBI and IB% decreased between 0–24 and 24–48 h (p = 0.001, and p = 0.001), but there were no further changes at 48–72 h. These changes were not seen in infants with poor outcome.

Thirty-five infants (13 with poor and 22 with good outcome) had normal cUS during the first 3 days. In this subgroup, infants with poor outcome had lower BG scores at 24–48 h (median; range: 3; 2–4 vs. 4; 2–5, p = 0.012) and 48–72 h (3; 2–5 vs. 4; 2–5, p = 0.039), longer IBI at 24–48 h (7.1; 4.4–9.7 vs. 5.1; 2.4–8.4 sec, p = 0.008), and higher IB% at 24–48 (59; 24–68 vs. 39; 22–69, p = 0.002) and 48–72 h (56; 26- 67 vs. 33; 14–68, p = 0.025) as compared to infants with good outcome. Studying survivors only, the 11 infants with NDI had lower BG scores at 24–48 h (median; range: 3; 2–4 vs. 4; 2–5, p = 0.019) and 48–72 h (3; 2–4 vs. 4; 2–5, p = 0.010), longer IBI at 24–48 h (6.1; 4.9-9.7 vs. 4.9; 2.4-8.4 sec, p = 0.013) and 48–72 h ( 6.5; 3.8–8.4 vs.4.5; 3.0–8.8) and higher IB% at 24–48 (56; 40-66 vs. 40; 16-68, p = 0.002) and 48–72 h (58; 26-66 vs. 33; 14-68, p = 0.019) as compared to the 30 infants without NDI.

### Seizures in aEEG/EEG

**Figure 4:**
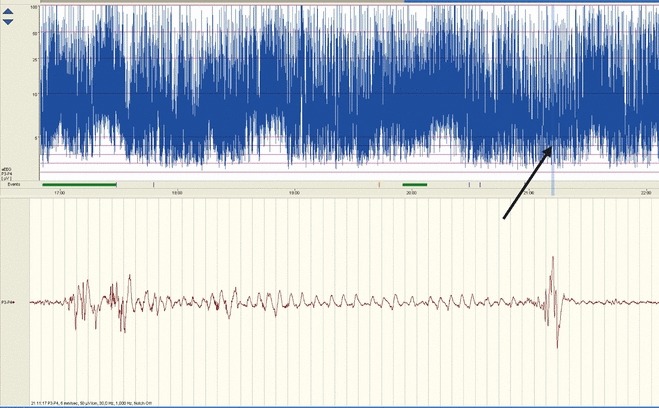
Four hours of amplitude-integrated electroencephalogram (aEEG) trend and below 54 sec of single-channel EEG corresponding to the arrow in the aEEG trend. A seizure pattern (duration 34 sec) is visible in the single-channel EEG but not in the aEEG trend. The aEEG trend shows a discontinuous pattern with immature cyclicity.

Electrographic seizures were present in 21 infants (43%). Altogether 89 seizures with duration from 12 sec to 6 h 10 min (median 60 sec) were identified (example in Figure 4). The total seizure duration per infant ranged from 20 sec to 16 h 56 min (median 4 min 14 sec). Status epilepticus was present in two infants with poor outcome; otherwise, there were no differences in seizure occurrence or total seizure duration per recorded hour between infants with good and poor outcome. Seizures were however more prevalent (65% vs. 31%, p = 0.024) among infants with brain injury during the first week according to cUS as compared to infants without cUS abnormalities. Forty-nine percent of the seizures (44 of 89) were retrospectively identified in the aEEG trend in 11 of the 21 infants who had seizures in original single-channel EEG; the median (range) duration of these seizures was 96 sec (25 sec–6 h 10 min).

### Quantification of a high-quality single-channel EEG at 24 h age

Interburst intervals and IB% differed significantly between infants with poor and good outcome in the 1-h high-quality recording obtained at 24 h (p = 0.003 and p < 0.001). EPT infants with poor outcome had longer IBI and higher IB% (Table 3). Among infants with normal cUS at day three, IBI and IB% were increased in infants with poor outcome compared to infants with good outcome (median; range: 7.3; 4.9–12.5 vs. 5.8; 2.3–17.7 sec, p = 0.04, and median; range: 61; 35–82 vs. 40; 0–82%, p = 0.004, respectively).

### Prediction of outcome

The prognostic value of aEEG/EEG features and cUS are summarized in Table 4. There were no significant differences between BG (presence of BS), SWC, IBI or IB%, respectively, as predictors of outcome. The highest proportion of correctly predicted outcomes in the overall study group was achieved using a cut-off limit for IB% at >55% (graphically represented and intuitively understood as an EEG where IBIs, that is, not burst activity, just slightly dominate the picture) in the 1-h recording (AUC 0.79 with 95% CI, 0.65–0.93). In multivariate regression models adjusting for GA, a 10%-unit increase in IB% was associated with an odds ratio of 2.2 [95% CI, 1.3–3.8] for poor outcome. In the subgroup of 35 infants with normal cUS during the first 3 days, 82% were correctly predicted by IB% at 24–48 h using the >55% cut-off. PPV, NPV, sensitivity and specificity were there 82%, 83%, 69% and 90%, respectively. In surviving infants, PPV, NPV, sensitivity, specificity and percentage of correctly predicted infants were 67%, 83%, 56%, 89% and 80% for prediction of NDI.

**Table 4:** Predictors of poor outcome (death or survival with neurodevelopmental impairment at 2 years corrected age) during the first 72 postnatal hours according to univariate analysis in 49 extremely preterm and very preterm infants with GA 22–30 weeks

Predictor	Sensitivity (%)	Specificity (%)	PPV (%)	NPV (%)	Percentage correctly predicted [95% CI]
IVH grade 1–4 or PVL on cUS	32	73	43	68	57 [38–75]
aEEG/EEG: burst suppression	89	67	63	91	76 [62–89]
aEEG: no continuous activity	89	53	54	89	63 [46–80]
aEEG/EEG: no cyclicity	58	77	61	74	69 [54–85]
EEG: seizures	44	43	40	47	34 [12–57]
EEG: median IBI > 6 sec in long-term monitoring 24–48 h	63	79	63	79	73 [57–88]
EEG: IB% > 55% in long-term monitoring 24–48 h	59	90	77	79	78 [65–92]
EEG: 1-h rec at 24 h: median IBI > 6 sec	67	79	67	79	74 [54–89]
EEG: 1-h rec at 24 h: IB% > 55%	72	83	72	80	79 [66–92]

aEEG = amplitude-integrated electroencephalogram; cUS = cerebral ultrasound investigations; GA = gestational age; IB% = interburst percentage; IBI = interburst intervals; IVH = intraventricular haemorrhages; NPV = negative predictive value; PPV = positive predictive value; PVL = periventricular leukomalacia.

## Discussion

Our study demonstrates that the electrocortical background activity, as recorded with single-channel aEEG/EEG, contains predictive information on long-term outcome in VPT infants already at 24 h of age, irrespective of brain damage according to cUS, and GA at birth. Overall, the outcome in around 80% of the infants could be correctly predicted by aEEG/EEG at 24 postnatal hours.

Neural dysfunction, reflected by aberrations in aEEG/EEG, may be a sign of developing brain damage. Early identification of such electrocortical dysfunction may thus open a time window for possible neuroprotective interventions. As compared to term asphyxiated infants (2), the prognostic value of the very early aEEG/EEG is clearly lower in VPT infants. One reason for this difference is that postnatal morbidity may be more important for the development of brain damage in preterm infants. For example, in our study, an infant of 25 weeks GA had low IB% and short IBIs in early EEG, indicating good outcome, but died from complications of necrotizing enterocolitis at 70 postnatal days. Still and according to the findings in the present study, we consider the predictive accuracy of the very early aEEG/EEG in EPT infants to be unexpectedly high, and indicating that early influence on brain function has an impact on long-term neurodevelopment in these infants.

The EEG measures IBI and IB% are directly related to the developmental neurobiology because they are based on identification of ‘bursts’, or ‘spontaneous activity transients’, which are considered important for brain wiring during development (16). In the present study, quantitative data based on IBI measurements and visual scoring of the aEEG/EEG background seem to provide comparable predictive information, and the combination of these measures did not increase the prognostic accuracy further.

We assessed the quality of the IBI and visually distinguished the isoelectric IBI during BS from tracé discontinu, the normal DC background activity of VPT EEG. It has been postulated that BS represents a functional thalamo-cortical disconnection in the mature brain (17). However, the significance of BS in VPT infants is not known although transient BS may appear after administration of, for example, morphine or in association with late onset sepsis (18). We found that transient BS, with duration more than two hours, is common in EPT infants and is associated with poor outcome. To our knowledge, the current study is the first to establish that electrocortical background activity with very low-voltage IBI, classified as BS, is associated with adverse outcome in EPT infants.

Artefacts are common in long-term aEEG/EEG monitoring in preterm infants, and therefore, we assessed the predictive value of quantitative data from a 1-h high-quality single-channel EEG obtained at 24 postnatal hours, also with at least a 4-h interval between the recording and administrated morphine. The predictive value of IB% was similar to the predictive value of visual and quantitative assessments in the long-term monitoring, which emphasizes the importance of high recording quality. Still, combined quantitative and visual assessment by an expert reader may be even better, as shown by West et al. (5). Also, the predictive value of the aEEG on long-term outcome was higher at 1–2 weeks in slightly more mature infants that underwent repeated aEEG recordings, demonstrating that the sensitivity for predicting adverse outcome was low and decreased for each week, while the specificity for a persistently abnormal pattern to predict poor outcome remained high (6).

Seizures in preterm infants are markers of abnormal brain function and carry an increased risk for abnormal outcomes (5,19,20). In previous studies, suspected seizures in the aEEG trend were associated with the development of IVH and illness severity (21,22). Shah et al. diagnosed seizures in the aEEG trend, with secondary confirmation by simultaneous two-channel EEG, in 51 preterm infants. Seizures were identified in 11 (22%) infants and were associated with adverse neonatal outcome (death or abnormal MRI) (23). In our study group, seizures were identified in 43% of the infants; they were more prevalent in infants with IVH, but their presence or duration was not associated with long-term outcome. Interestingly, if seizures had been identified only in the aEEG, 22% of our study cohort (11/49) would have had been diagnosed with seizures, very similar to the rate of seizure detection reported by Shah et al.

Electroencephalogram background activity increases during the first postnatal days in preterm infants (20,22,24,25). In the present study, we found increasing activity during the first 48 h, but only in infants with good outcome. It could be expected that the more mature VPT infants should have higher aEEG/EEG scores and lower IBI and IB% than EPT infants with good outcome, and as no such difference was seen during the first 24 h but at 24–48 h age, early postnatal recovery may explain the increasing activity.

In conclusion, our data show that cerebral depression as assessed by early aEEG/EEG is associated with long-term outcome in VPT and EPT infants. The relatively small study population is a limitation, and the findings need to be reproduced in larger cohorts. We included infants up to 30 gestational weeks but focused on EPT infants below 28 gestational weeks, because these infants have the highest risks for subsequent brain damage. Both visual pattern recognition and quantitative analysis of the aEEG/EEG background provide long-term predictive information in VPT and EPT infants. Brief subclinical seizures are common during the first days and are associated with IVH, but not to long-term outcome.
